# Testosterone and sex hormone-binding globulin in dysglycemic women at high cardiovascular risk: A report from the Outcome Reduction with an Initial Glargine Intervention trial

**DOI:** 10.1177/14791641211002475

**Published:** 2021-03-22

**Authors:** Anne Wang, Hertzel C Gerstein, Shun Fu Lee, Sibylle Hess, Guillaume Paré, Lars Rydén, Linda G Mellbin

**Affiliations:** 1 Department of Medicine Solna, Karolinska Institutet, Stockholm, Sweden; 2 Population Health Research Institute, McMaster University and Hamilton Health Sciences, Hamilton, ON, Canada; 3 Department of Medicine, McMaster University, Hamilton, ON, Canada; 4 R&D, Translational Medicine & Early Development, Biomarkers & Clinical Bioanalyses, Sanofi Aventis Deutschland GmbH, Frankfurt, Germany; 5 Heart and Vascular Theme, Karolinska University Hospital, Stockholm, Sweden

**Keywords:** Cardiovascular, diabetes, prognosis, sex hormone-binding globulin, testosterone, women

## Abstract

**Aims:**

Total and free testosterone and sex hormone-binding globulin may affect cardiovascular prognosis in women. The objective was to study the association between sex hormones and prognosis in women with dysglycemia and high cardiovascular risk.

**Methods:**

This epidemiological report included dysglycemic women from the Outcome Reduction with an Initial Glargine Intervention trial (*n* = 2848) with baseline total testosterone and sex hormone-binding globulin. Free testosterone was calculated with the Vermeulen formula. Cox regression analyses adjusted for variables including age, previous diseases and pharmacological treatments were used to estimate the association between these levels and the composite cardiovascular outcome (death from cardiovascular causes, nonfatal myocardial infarction or nonfatal stroke) and all-cause mortality per one standard deviation.

**Results:**

Patients (73% post-menopausal) were followed for a median of 6.1 years during which 377 cardiovascular events and 389 deaths occurred. In Cox analyses, total and free testosterone were not associated with any outcomes, but sex hormone-binding globulin was related to all-cause mortality in age adjusted (HR 1.15; 95% CI 1.06–1.24; *p* < 0.01) and fully adjusted analyses (HR 1.14; 95% CI 1.05–1.24; *p* < 0.01).

**Conclusions:**

Increasing levels of baseline sex hormone-binding globulin were associated with an increased risk of all-cause mortality in dysglycemic women at high cardiovascular risk.

**Trial registration:**

ClinicalTrials.gov no. NCT00069784.

## Introduction

The importance of sex hormones in the development of cardiovascular disease in women has been debated. Whereas the focus has traditionally been on estrogen,^1^ the importance of testosterone and its binding protein sex hormone-binding globulin (SHBG) has attracted recent attention.^2^ Testosterone acts both as an androgen and as a precursor of estradiol and exerts important physiological effects on well-being, sexual function, bone metabolism and cardiometabolic health.^3^ Studies in women of reproductive age and with polycystic ovarian syndrome (PCOS), a condition typically characterized by hyperandrogenism (high testosterone levels) and hyperinsulinemia, have suggested an increased risk of developing cardiovascular risk factors however the effects on cardiovascular mortality is unclear.^4,5^ To what extent testosterone is related to prognosis in middle-aged and elderly women with dysglycemia remains to be clarified.

The level of free testosterone is in part regulated by SHBG. High levels of the latter can result in lower fractions of free testosterone. Whereas some observational studies have reported that cardiovascular disease, all-cause mortality and diabetes are associated with low levels of total and free testosterone as well as SHBG^6–8^ others have related some of these outcomes to high levels.^2,9,10^ These inconsistent results illustrate the importance of further research to elucidate the relationship between androgens and cardiovascular disease in women.

The present study investigates the prognostic impact of testosterone and SHBG on the development of cardiovascular outcomes in female participants in the Outcome Reduction with an Initial Glargine Intervention (ORIGIN) trial^11^ comprising a well-defined cohort of dysglycemic patients at high cardiovascular risk.

## Material and methods

As previously published, participants in ORIGIN comprised 12,537 men (65%) and women (35%) ⩾50 years with either prediabetes (impaired fasting glucose or impaired glucose tolerance) or type 2 diabetes and additional cardiovascular risk factors. They were randomly assigned to insulin glargine (Gla-100) *vs.* standard care and either omega 3 fatty acids *vs.* placebo using a 2 × 2 factorial design.^11,12^ Exclusion criteria included serious comorbid conditions such as active cancer, hepatic cirrhosis, chronic or recurrent treatment with systemic corticosteroids or an expected survival of less than 3 years by non-cardiovascular causes. During a median follow up time of 6.1 years, patients were followed for a composite outcome of cardiovascular events including cardiovascular death, non-fatal myocardial infarction or non-fatal stroke.^11,12^ A secondary outcome was all-cause mortality. Research Ethics Boards approved the ORIGIN trial in all study centres.

A subset of participants also consented to the collection and storage of blood samples for future measurement of cardiovascular risk factors. They were included in a biomarker study in which 8494 participants had biomarkers measured as outlined below and following elimination of unanalyzable and undetectable biomarkers, the total population was comprised of 8401 participants (34% women).^13^ The present study comprises female participants of this subset in whom total testosterone and SHBG were assayed (*n* = 2848).

### Laboratory analyses

Fasting blood samples at baseline were collected, divided into aliquots and transported to the Population Health Research Institute biobank in Hamilton for storage in nitrogen vapor-cooled tanks at −160°C. Following completion of the ORIGIN trial, coded aliquots of serum from each study participant were transported to Myriad RBM Inc (Austin, Texas). In a biomarker substudy, multiplex analysis of a predefined panel of 284 biomarkers was performed using a customized Human Discovery Multi-Analyte Profile (MAP) 250 + panel on the LUMINEX 100/200 platforms. The biomarker results were carefully scrutinized, leaving a total of 237 reported biomarkers from 8401 participants in the final dataset. This included total testosterone, SHBG and luteinizing hormone (LH) which had inter-run coefficients of variation at intermediate concentrations at 7%, 14% and 6% respectively.^13^ Estradiol was not available in the database.

### Definitions

*Free testosterone* was calculated according to the Vermeulen et al.^14^ formula with a fixed albumin concentration of 43 g/L.

### Statistical analyses

Continuous variables were presented as means (standard deviation [SD]) and compared using t-test, categorical variables were presented as numbers and percentage (%) and compared using chi-square test. The relationship between levels and outcomes were estimated using Cox proportional hazards regression models. Hazard ratios (HRs) and 95% confidence intervals (CI) were estimated per an increase of one SD for continuous variables. In multivariate models, Model A was adjusted for age; Model B was further adjusted for log-transformed LH levels (which regulates the production of testosterone), previous cardiovascular disease, previous diabetes, use of metformin, use of statins, systolic blood pressure, HbA1c, low-density lipoprotein (LDL) cholesterol, body mass index and smoking; and Model C was further adjusted for menopausal status, hormone treatment, alcohol consumption, ethnicity, obstructive sleep apnea and thyroid drugs. Whether the estimates differed according to allocated treatment group or previously established CVD was assessed by testing for interactions. Previously established CVD was defined as prior MI, stroke, revascularisation, or angina with documented ischemia compared to high CV risk which was defined as morning urinary albumin/creatinine ratio >30 µg/mg, evidence of left ventricular hypertrophy, 50% stenosis of a coronary, carotid or lower extremity artery documented angiographically and/or an ankle/brachial index <0.9. A two-sided *p*-value <0.05 was used as a nominal level of significance. Statistical analyses were performed using SAS version 9.4 (SAS Institute Inc, Cary, NC).

## Results

### Clinical characteristics

Baseline characteristics for the study cohort are outlined in Table 1. A total of 2848 women of mean age 64 years whereof 73% were post-menopausal with either prediabetes (16%) or diabetes (84%) were included. Previous cardiovascular events (including myocardial infarction, stroke and revascularisation) were reported by 43%, and 23% reported a previous myocardial infarction. Mean (SD) BMI was 31.1 (6.2) kg/m^2^, mean (SD) total cholesterol was 5.2 (1.2) mmol/L and mean (SD) HbA1c was 6.6 (1.0)% (IFFS 49 mmol/mol). Statins were used in 44% of the patients, aspirin in 56% and ACE-inhibitors/ARB in 69%.

**Table 1.** Baseline characteristics of the study participants. Continuous variables presented as mean (standard deviation) and categorical variables presented as *n* (%).

	All participants *n* = 2848
Clinical characteristics	
Age (years)	64.0 (8.0)
Known diabetes	2399 (84.2)
Newly detected diabetes	150 (5.3)
Newly detected IGT/IFG	298 (10.5)
Diabetes duration (years)	5.4 (5.9)
Previous cardiovascular events	1228 (43.1)
Myocardial infarction	640 (22.5)
Hypertension	2419 (84.9)
Smoker	296 (10.4)
Alcohol consumption >2 drinks/week	218 (7.7)
Menopause	2086 (73.2)
Menopause + Hormone therapy	102 (3.6)
Thyroid hormone treatment	319 (11.2)
Obstructive sleep apnea	71 (2.5)
Pharmacological treatment	
Metformin	886 (31.1)
Sulfonylurea	810 (28.4)
Other glucose lowering drug	51 (1.8)
No glucose lowering drug	1101 (38.7)
Statin	1241 (43.6)
ACE inhibitors/ARB	1957 (68.7)
Beta-blockers	1366 (48.0)
Thiazide diuretics	691 (24.3)
Aspirin	1597 (56.1)
Other antiplatelet drugs	265 (9.3)
Laboratory findings at baseline	
eGFR (mL/min/1.73 m^2^)	74.5 (22.6)
Body weight (kg)	77.9 (16.9)
BMI (kg/m^2^)	31.1 (6.2)
Systolic blood pressure (mmHg)	148.4 (22.7)
HbA1c (DCCT %; mmol/mol)	6.6 (1.0); 49
Cholesterol (mmol/L)	5.2 (1.2)
HDL-cholesterol (mmol/L)	1.3 (0.3)
LDL-cholesterol (mmol/L)	3.1 (1.1)
Triglycerides (mmol/L)	1.9 (1.1)
Total testosterone (ng/dL)	122.6 (76.6)
Free testosterone (ng/dL)	2.2 (1.7)
SHBG (nmol/L)	44.6 (24.7)
LH (mIU/L)	8.4 (4.1)

ACE/ARB: Angiotensin Converting Enzyme/Angiotensin Receptor II Blocker; BMI: Body Mass Index; eGFR: Estimated glomerular filtration rate; HbA1c: glycosylated haemoglobin; HDL: High Density Lipoprotein; IGT: Impaired glucose tolerance; IFG: Impaired fasting glucose; LDL: Low Density Lipoprotein; LH: Luteinizing hormone; SHBG: Sex hormone-binding globulin.

To convert testosterone from ng/dL to nmol/L, multiply by 0.0347.

The mean (SD) of total and free testosterone levels were 122.6 (76.6) ng/dL and 2.2 (1.7) ng/dL respectively. The mean (SD) SHBG levels were 44.6 (24.7) nmol/L. There was no difference in testosterone or SHBG levels between those with or without previously established CVD (data not shown).

### Testosterone, SHBG and prognosis

**Figure 1.**
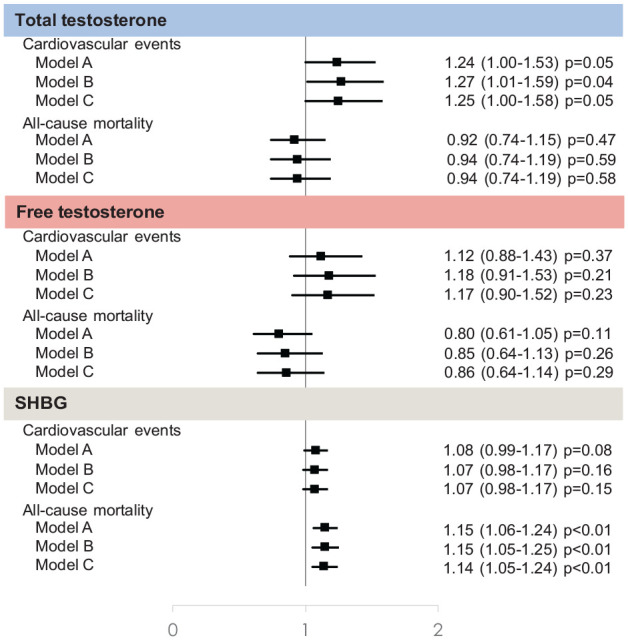
Hazard ratio (95% CI) for the association between sex hormones and cardiovascular events and all-cause mortality by increase of one standard deviation. Model A adjusted for age. Model B adjusted for age, luteinizing hormone (log and standardized), previous cardiovascular disease, previous diabetes diagnosis, use of metformin, use of statins, systolic blood pressure, HbA1c, low-density lipoprotein cholesterol, body mass index and smoking. Model C adjusted for Model B plus menopause and no hormone therapy, menopause and hormone therapy, thyroid hormone treatment, alcohol consumption, white ethnicity, obstructive sleep apnea.

There were 377 cardiovascular events and 389 all-cause deaths during a median follow-up of 6.1 years (IQR: 5.8–6.6) in the female subgroup. The concentration of total testosterone was not significantly associated with cardiovascular events after adjustment for age (Model A) (HR 1.24, 95% CI 1.00–1.53; *p* = 0.05) and after adjustment for 16 other cardiovascular risk factors (Model C) (HR 1.25, 95% CI 1.00–1.58; *p* = 0.05; Figure 1). There was a nominally significant association with cardiovascular events after adjustment for 10 cardiovascular risk factors (Model B) (HR 1.27, 95% CI 1.01–1.59; *p* = 0.04). There was no evidence of a relationship between total testosterone levels and all-cause mortality.

The concentration of free testosterone was not significantly associated with either cardiovascular events or all-cause mortality (Figure 1).

The concentration of SHBG was not significantly associated with cardiovascular events (Figure 1). However each SD increase was related to a 1.15 times higher hazard of all-cause mortality in the age adjusted model (HR 1.15, 95% CI 1.06–1.24; *p* < 0.01), after further adjustment (HR 1.15, 95% CI 1.05–1.25; *p* < 0.01) and after maximal adjustment (HR 1.14, 95% CI 1.05–1.24; *p* < 0.01).

There were no significant interactions between hormone levels and study allocations or previously established CVD (Supplemental Table S1).

## Discussion

The analysis of 2848 middle-aged and elderly women in which the majority had reached menopause and with dysglycemia and high cardiovascular risk identified baseline high SHBG as a predictor for future death during a median follow up of 6.1 years.

The current literature on the prognostic capacity of testosterone and SHBG in women is inconclusive, with some studies suggesting a U-shaped relationship with the implication that those with the lowest or the highest levels have an impaired survival^15,16^ while other investigations are devoid of any prognostic association.^17–20^ The main results from a recent report from the Multi-Ethnic Study of Atherosclerosis (MESA; *n* = 2834 postmenopausal women) showed that higher total testosterone was associated with increased risk of cardiovascular disease. A similar trend, however with nominal significance, was seen in the present study. Interestingly, higher SHBG but not testosterone was associated with all-cause mortality. The prognostic effect of SHBG is in part supported by other studies^6,16,21^ and in a subgroup analysis from the MESA report excluding women on hormone therapy (*n* = 1934), high levels of SHBG and low levels of free testosterone and estradiol were related to an elevated risk of coronary heart disease.^9^ The present lack of an association between the hormones and cardiovascular disease may relate to the discrepant study populations. The MESA study recruited women free from cardiovascular disease whereas the present study included dysglycemic women either at high cardiovascular risk or with previous cardiovascular events. It may be that in patients already afflicted with glucose perturbations and cardiovascular disease there are more important factors than sex hormones to predict future cardiovascular events, as has been shown before.^7,22^

A possible explanation for the association between SHBG and all-cause mortality relates to the biological interplay between these hormones. SHBG binds most of circulating sex hormones such as testosterone and estradiol leaving only a small portion circulating freely and affecting tissues.^23^ Thus, high levels of SHBG may result in lower levels of free, bioactive sex hormones. This suggest that SHBG may be seen as a marker of bioavailable testosterone, which may be a partial explanation to its link to all-cause mortality in this group of dysglycemic women. However, the lack of a distinct predictive ability of testosterone in the present study suggests that other, testosterone independent pathways should also be considered. SHBG levels are altered in conditions such as diabetes,^23^ in part due to high insulin levels inhibiting SHBG production.^24^ During the process of progression from dysglycemia to diabetes, high insulin levels are usually found early and fall with time. This may lead to higher levels of SHBG. Thus, one hypothesis is that a rise in SHBG may reflect a fall in insulin secretion and progression of dysglycemia, which might explain its prognostic implications. To what extent the possible link between SHBG and insulin can explain the prognostic ability of SHBG cannot be derived from the present data, but underlines the need for clarifying the mechanism of action for SHBG as a predictor of mortality in dysglycemic women.

### Strengths and weaknesses

The present study is based on a large, well-defined population with detailed information on menopausal status and hormone therapy. Patients were followed for a long period of time and all events were prospectively collected. Still there are some potential limitations to be considered. Testosterone was estimated by use of the LUMINEX 100/200 platforms with measurement of several biomarkers at the same time, rather than mass spectrometry, which is considered the most sensitive method. Free testosterone was not measured directly but, in line with current guidelines,^25^ calculated using the well established algorithm of Vermeulen et al. Moreover, although the prevalence of PCOS is likely to be low, information on this was not gathered and could have contributed to higher mean testosterone levels in the present compared to previous studies. Lastly, the observations were based on a single sample of sex hormone levels obtained at baseline.

## Conclusion

In conclusion, high SHBG in middle-aged and elderly women with different levels of dysglycemia and at high cardiovascular risk is related to an increased risk of all-cause mortality and high total testosterone was nominally associated with cardiovascular events. This highlights the importance of further studies regarding the prognostic role of androgens in females.

## Supplemental Material

sj-pdf-1-dvr-10.1177_14791641211002475 – Supplemental material for Testosterone and sex hormone-binding globulin in dysglycemic women at high cardiovascular risk: A report from the Outcome Reduction with an Initial Glargine Intervention trial Click here for additional data file. Supplemental material, sj-pdf-1-dvr-10.1177_14791641211002475 for Testosterone and sex hormone-binding globulin in dysglycemic women at high cardiovascular risk: A report from the Outcome Reduction with an Initial Glargine Intervention trial by Anne Wang, Hertzel C Gerstein, Shun Fu Lee, Sibylle Hess, Guillaume Paré, Lars Rydén and Linda G Mellbin in Diabetes & Vascular Disease Research
