# Supplementary material for: Testosterone and sex hormone-binding globulin in dysglycemic women at high cardiovascular risk: A report from the Outcome Reduction with an Initial Glargine Intervention trial
Source: Diab Vasc Dis Res. 2021 Mar 22;18(2):14791641211002475. doi: 10.1177/14791641211002475 (PMC8481727; doi:10.1177/14791641211002475)
Supplement: sj-pdf-1-dvr-10.1177_14791641211002475 – Supplemental material for Testosterone and sex hormone-binding globulin in dysglycemic women at high cardiovascular risk: A report from the Outcome Reduction with an Initial Glargine Intervention trial [file sj-pdf-1-dvr-10.1177_14791641211002475.pdf]

Supplementary table 1

Table S1: Interactions analyses for sex hormones with cardiovascular events and all-cause mortality by study allocation and previously established CVD*.

	Cardiovascular events		All-cause mortality	
Variable	Estimate	p	Estimate	p
By study allocation[a]				
Total testosterone	0.0000	0.9903	-0.0013	0.3475
Free testosterone	0.0171	0.7848	-0.0541	0.4366
SHBG levels	0.0017	0.6859	0.0012	0.7575
By previously established CVD[b]				
Total testosterone	0.0023	0.0881	0.0015	0.2822
Free testosterone	0.0902	0.1555	0.1078	0.1211
SHBG levels	-0.0012	0.7696	-0.0025	0.5274

*defined as previous MI, stroke or revascularization, or angina with documented ischemia.
[a] Model B adjusted for glargine allocation interaction with variable of interest
[b] Model B adjusted for previously established CVD interaction with variable of interest
